# RETRACTION: Sevoflurane Ameliorates Myocardial Cell Injury by Inducing Autophagy via the Deacetylation of LC3 by SIRT1

**DOI:** 10.1155/ancp/9862018

**Published:** 2026-06-10

**Authors:** Analytical Cellular Pathology

RETRACTION: L. Fan, D. Chen, J. Wang, Y. Wu, D. Li, and X. Yu, “Sevoflurane Ameliorates Myocardial Cell Injury by Inducing Autophagy via the Deacetylation of LC3 by SIRT1,” *Analytical Cellular Pathology* 2017, no. 1 (2017): 6281285, https://doi.org/10.1155/2017/6281285.

The above article, published online on 26 September 2017 in Wiley Online Library (wileyonlinelibrary.com), has been retracted by agreement between the Journal Editor‐in‐Chief, Dimitrios Karamichos; and John Wiley & Sons Ltd. The retraction has been agreed after an investigation into image concerns raised by René Aquarius via PubPeer [1]. Specifically, the ‘NG’ and ‘MG’ panels in Figure 3 share unexpected similarities. Further investigation also revealed suspected image manipulation involving the Western blots within Figures 6 and 7.

The authors were unresponsive to the journal’s attempts to contact them regarding these concerns. As such, the Editor no longer considers the results to be reliable, and the article is therefore retracted.
